# Comparative burden and projections of chewing tobacco-attributable lip/oral cavity and esophageal cancers: global and China-specific trends, 2000–2036

**DOI:** 10.1080/07853890.2026.2635110

**Published:** 2026-02-25

**Authors:** Zhisheng Teng, Jiao Pang, Chao Chen

**Affiliations:** a Department of Head and Neck Surgery, Zhejiang Cancer Hospital, Hangzhou Institute of Medicine (HIM), Chinese Academy of Sciences, Hangzhou, China; b College of Life Science, Northwest University, Xi’an, China

**Keywords:** Burden of disease, epidemiology, projection, chewing tobacco, lip and oral cavity cancer, esophageal cancer

## Abstract

**Background:**

Smokeless tobacco (SLT), particularly chewing tobacco, is an underrecognized public health concern. Its long-term burden and trends, especially in China, remain incompletely quantified.

**Methods:**

Using Global Burden of Disease (GBD) 2021 data, we estimated chewing tobacco–attributable deaths, disability-adjusted life years (DALYs), and age-standardized mortality and DALY rates for lip and oral cavity cancer and esophageal cancer (2000–2021), globally and in China. Analyses were stratified by year, sex, and age. Decomposition, age–period–cohort (APC), and Bayesian age–period–cohort (BAPC) models assessed drivers and project trends.

**Results:**

From 2000 to 2021, chewing tobacco–attributable lip and oral cavity cancer deaths and DALYs nearly doubled globally and in China, with modest rises in age-standardized rates. Esophageal cancer showed slight absolute increases but declining standardized rates. For both cancers, DALYs peaked earlier than deaths. Compared with global patterns, China experienced a steeper increase in age-standardized lip and oral cavity cancer burden, particularly among males, and a larger decline in esophageal cancer burden, especially among females, leading to increasing male predominance. Globally, changes mainly reflected population growth and aging, whereas population growth predominated in China. Projections indicate continued increases in lip and oral cavity cancer burden and further declines in esophageal cancer burden.

**Conclusions:**

Chewing tobacco–attributable lip and oral cavity cancer burden in China has risen markedly and is projected to increase further, particularly among males and working-age populations, whereas esophageal cancer burden continues to decline. Integrated prevention strategies are needed to sustain progress and reduce the growing burden.

## Introduction

Tobacco use is one of the most preventable cause of numerous health diseases and disorders [1], resulting in approximately 443,000 deaths annually and accounting for at least 30% of cancer-related mortality [2]. For lip and oral cavity cancer and esophageal cancer, the relative attributable risk of tobacco use has been estimated at 60%–70% [3–5]. With stricter smoking regulations, there has been a noticeable increase in the consumption of smokeless tobacco (SLT) [6]. Chewing tobacco, the most common form of SLT, therefore represents an important yet often underaddressed exposure in international public health research and tobacco control policies [7].

Lip and oral cavity cancer, classified within head and neck cancers, ranks 16th in incidence and 15th in mortality among all cancer types globally [8]. Esophageal cancer is a prevalent malignancy, ranking 7th in incidence and 6th in mortality worldwide [9]. Lip and oral cavity cancer is prevalent in numerous developing nations, particularly with a significant disease burden in China [10]. Notably, China has one of the highest burdens of esophageal cancer worldwide, which accounts for approximately 50% of global incidence and death cases annually [11].

Despite the established carcinogenic role of chewing tobacco, few studies have systematically compared long-term trends or projected future trajectories of chewing tobacco–attributable lip and oral cavity cancer and esophageal cancer across countries and within China—the world’s largest tobacco consumer [12].

Using estimates from the Global Burden of Disease (GBD) Study 2021, we quantified chewing tobacco–attributable deaths, disability-adjusted life years (DALYs), and age-standardized rates for both cancers. We further applied decomposition analysis, age–period–cohort analysis, and BAPC projection analysis to assess risk factors, age–period–cohort effects, and future trends. By comparing global and national patterns, especially in China, this study aims to inform temporal, site-specific, sex-specific, age-specific, and cancer-specific prevention strategies and to strengthen the evidence base for tobacco-related cancer control policies.

## Materials and methods

Raw data were obtained from the GBD Study Results Tool (http://ghdx.healthdata.org/gbd-results-tool), which provides comprehensive, publicly accessible estimates of disease burden attributable to specific risk factors across 204 countries and territories [13]. We focused on lip and oral cavity cancer and esophageal cancer attributable to chewing tobacco use. Data were extracted for deaths, disability-adjusted life years (DALYs), age-standardized mortality rates (ASMRs), and age-standardized DALY rates (ASDRs). All age-standardized rates were directly obtained from the GBD 2021 Results Tool and calculated using the GBD 2013 global age-standard population (hybrid) as the reference population. Estimates were stratified by sex, age, time, site, and cancer type.

DALYs were calculated as the sum of years of life lost (YLLs) and years lived with disability (YLDs) [12]. Age-standardized rates (ASRs), including ASMRs and ASDRs, were used to facilitate comparisons across populations with differing age structures [14]. All rates were expressed per 100,000 population. Age-specific analyses were restricted to individuals aged ≥30 years, consistent with the GBD framework and the negligible burden of chewing tobacco–attributable lip and oral cavity cancer and esophageal cancer at younger ages. Age groups were categorized in 5-year intervals from 30–34 to 95+ years to describe age-specific burden patterns. Male-female ratios were calculated to assess gender disparities [15].

Temporal trends between 2000 and 2021 were quantified using the estimated annual percentage change (EAPC), derived from log-linear regression of ASRs against calendar year [16]. The equation was specified as [17]:

$$\text{ln}\left(\text{ASR}\right)=\alpha +\beta X+\varepsilon$$

where X represents the calendar year, β denotes the annual rate of change, and ε is the random error term. The EAPC was calculated as [18]:

$$\text{EAPC}=100\;\times \;\left(\text{exp}\left(\beta \right)-1\right)$$

An upward trend is recognized when both the estimated EAPC and the lower limit of the 95% confidence interval (CI) were greater than zero [19]. A downward trend is established if both the estimated EAPC and the upper limit of the 95% CI were less than zero [20]. Otherwise, trends were considered stable, in accordance with standard GBD trend classification criteria [16,21]. The Human Development Index (HDI) was used as an indicator of socioeconomic development when interpreting cross-national differences in disease burden [22].

Decomposition analysis was performed to quantify the contributions of population growth, population aging, and epidemiological changes to chewing tobacco-attributable deaths and DALYs. Epidemiological change represents the residual variation after accounting for demographic effects and reflects non-demographic factors, including changes in risk-factor exposure, disease incidence, case fatality, and health-system performance [23]. Age–period–cohort (APC) analysis was conducted to evaluate the independent effects of age, period, and birth cohort on deaths and DALYs. Ratio rates (RRs) were estimated using 1990–1995 as the reference period and 1960 as the reference birth cohort. Future trends were projected using a BAPC prediction model, implemented with the ‘nordpred’ (version 1.1), ‘BAPC’ (version 0.0.36) and ‘INLA’ (version 24.6.27) packages in R. Within the BAPC framework, age, period, and cohort effects were assigned second-order random-walk priors (RW2) to ensure smooth temporal variation. All statistical analyses and data visualization of the data were implemented in R (version 4.4.2), with statistical significance defined as *p* < 0.05. To account for uncertainty, GBD-recommended estimates are presented with 95% uncertainty intervals (UIs), and sensitivity analyses were conducted to assess the robustness of the overall findings.

## Results

### Burdens of chewing tobacco–attributable lip and oral cavity cancer and esophageal cancer worldwide and in China from 2000 to 2021

Globally, from 2000 to 2021, the number of chewing tobacco-attributable deaths from lip and oral cavity cancer almost doubled, rising from 20,846.9 to 38,903.6. Despite this rise, the ASMR exhibited relative stability, rising slightly from 0.417 per 100,000 in 2000 to 0.450 in 2021 (EAPC: 0.348, 95% CI: 0.261–0.436). The upward trend in ASMR was more significant in females (EAPC: 0.614, 95% CI: 0.491–0.737) than males (EAPC: 0.037, 95% CI: −0.046–0.12) (Table 1). The burden of DALYs exhibited a similar pattern, increasing from 621,796.4 to 1,102,818.4, with ASDR rising slightly from 11.849 to 12.651 per 100,000 (EAPC: 0.299, 95% CI: 0.197–0.400) (Table 2). For esophageal cancer, the absolute number of deaths increased slightly from 12,214.7 to 17,939.5, but the ASMR decreased from 0.246 to 0.207 per 100,000 (EAPC: −0.966, 95% CI: −1.190 to −0.823). This downward trend was observed in both sexes, with a steeper decline among males (EAPC: −1.045, 95% CI: −1.211 to −0.878) than females (EAPC: −0.921, 95% CI: −1.03 to −0.813) (Table 1). DALYs similarly increased from 347,853.9 to 479,032.7, with ASDR falling from 6.672 to 5.461 per 100,000 (EAPC: −1.115, 95% CI: −1.269 to −0.960) (Table 2).

**Table 1.** Deaths numbers, age-standardized mortality rates (ASMRs), and estimated annual percentage changes (EAPCs) of lip and oral cavity cancer and esophageal cancer attributable to chewing tobacco worldwide and in China, 2000–2021.

	Characters	2000		2021		2000–2021
		Deaths numbers	ASMRs (per 100,000)	Deaths numbers	ASMRs (per 100,000)	EAPCs (95% CI)
Global	Lip and oral cavity cancer					
	Both	20,846.895	0.417	38,903.648	0.450	0.348 (0.261, 0.436)
	Male	11,112.295	0.468	19,792.834	0.480	0.037 (−0.046, 0.120)
	Female	9,734.600	0.370	19,110.815	0.416	0.614 (0.491, 0.737)
	Esophageal cancer					
	Both	12,214.710	0.246	17,939.524	0.207	−0.966 (−1.109, −0.823)
	Male	8,218.840	0.355	12,087.829	0.297	−1.045 (−1.211, −0.878)
	Female	3,995.870	0.152	5,851.695	0.127	−0.921 (−1.030, −0.813)
China	Lip and oral cavity cancer					
	Both	173.701	0.016	370.945	0.018	0.978 (0.715, 1.241)
	Male	97.460	0.018	225.162	0.022	1.751 (1.463, 2.041)
	Female	76.240	0.014	145.783	0.013	−0.001 (−0.470, 0.471)
	Esophageal cancer					
	Both	1,888.092	0.168	2,442.009	0.115	−2.223 (−2.574, −1.872)
	Male	1,556.343	0.277	2,067.625	0.203	−1.876 (−2.192, −1.558)
	Female	331.749	0.063	374.384	0.034	−3.485 (−3.980, −2.988)

The table presents the numbers of deaths and ASMRs (per 100,000 population) in 2000 and 2021, stratified by sex, for lip and oral cavity cancer and esophageal cancer attributable to chewing tobacco. Temporal trends from 2000 to 2021 are summarized using EAPCs with 95% confidence intervals (CIs). ASMRs were calculated using the Global Burden of Disease standard population.

**Table 2.** Disability-adjusted life years (DALYs) numbers, age-standardized DALY rates (ASDRs), and estimated annual percentage changes (EAPCs) of lip and oral cavity cancer and esophageal cancer attributable to chewing tobacco worldwide and in China, 2000–2021.

	Characters	2000		2021		2000–2021
		DALYs numbers	ASDRs (per 100,000)	DALYs numbers	ASDRs (per 100,000)	EAPCs (95% CI)
Global	Lip and oral cavity cancer					
	Both	621,796.44	11.849	1,102,818.40	12.651	0.299 (0.197, 0.400)
	Male	348,538.30	13.549	602,349.12	14.213	0.162 (0.092, 0.232)
	Female	273,258.14	10.192	500,469.27	11.054	0.434 (0.262, 0.607)
	Esophageal cancer					
	Both	347,853.85	6.722	479,032.71	5.461	−1.115 (−1.269, −0.960)
	Male	240,802.77	9.624	332,862.05	7.882	−1.119 (−1.291, −0.947)
	Female	107,051.08	4.026	146,170.65	3.203	−1.130 (−1.254, −1.006)
China	Lip and oral cavity cancer					
	Both	5,445.912	0.449	10,708.774	0.510	1.108 (0.839, 1.376)
	Male	3,269.385	0.520	6,944.205	0.669	1.771 (1.497, 2.044)
	Female	2,176.527	0.372	3,764.569	0.348	0.070 (−0.429, 0.571)
	Esophageal cancer					
	Both	57,972.456	4.768	65,752.685	3.043	−2.548 (−2.947, −2.148)
	Male	49,777.688	7.998	57,825.100	5.443	−2.222 (−2.589, −1.854)
	Female	8,194.768	1.462	7,927.585	0.704	−4.017 (−4.579, −3.451)

The table presents the numbers of DALYs and ASDRs (per 100,000 population) in 2000 and 2021, stratified by sex, for lip and oral cavity cancer and esophageal cancer attributable to chewing tobacco. Temporal trends from 2000 to 2021 are summarized using EAPCs with 95% confidence intervals (CIs). ASDRs were calculated using the Global Burden of Disease standard population.

In China, the trends are similar between cancers. For lip and oral cavity cancer, chewing tobacco–attributable deaths rose from 173.7 to 370.9, and ASMR increased from 0.016 to 0.018 per 100,000 (EAPC: 0.978, 95% CI: 0.715–1.241), which is faster than global trend. This upward trend was more pronounced among males (EAPC: 1.751, 95% CI: 1.463–2.041) than females (EAPC: −0.001, 95% CI: −0.47–0.471) (Table 1). DALYs nearly doubled from 5,445.9 to 10,708.8, with ASDR increasing from 0.449 to 0.510 per 100,000 (EAPC: 1.108, 95% CI: 0.839–1.376) (Table 2). Conversely, chewing tobacco–attributable esophageal cancer showed a pronounced reduction. Although deaths rose slightly from 1,888.1 to 2,442.0, ASMR dropped sharply from 0.168 to 0.115 per 100,000 (EAPC: −2.223, 95% CI: −2.574 to −1.872), which is faster than the global rate. This decline was consistent across genders, but males showed a slight reduction (EAPC: −1.876, 95% CI: −2.192 to −1.558) compared to a steeper reduction for females (EAPC: −3.485, 95% CI: −3.980 to −2.988) (Table 1). DALYs increased from 57,972.5 to 65,752.7, yet ASDR plummeted from 4.768 to 3.313 per 100,000 (EAPC: −2.548, 95% CI: −2.947 to −2.148) (Table 2).

### Burdens of chewing tobacco–attributable lip and oral cavity cancer and esophageal cancer across both sexes in 204 countries and territories

**Figure 1.**
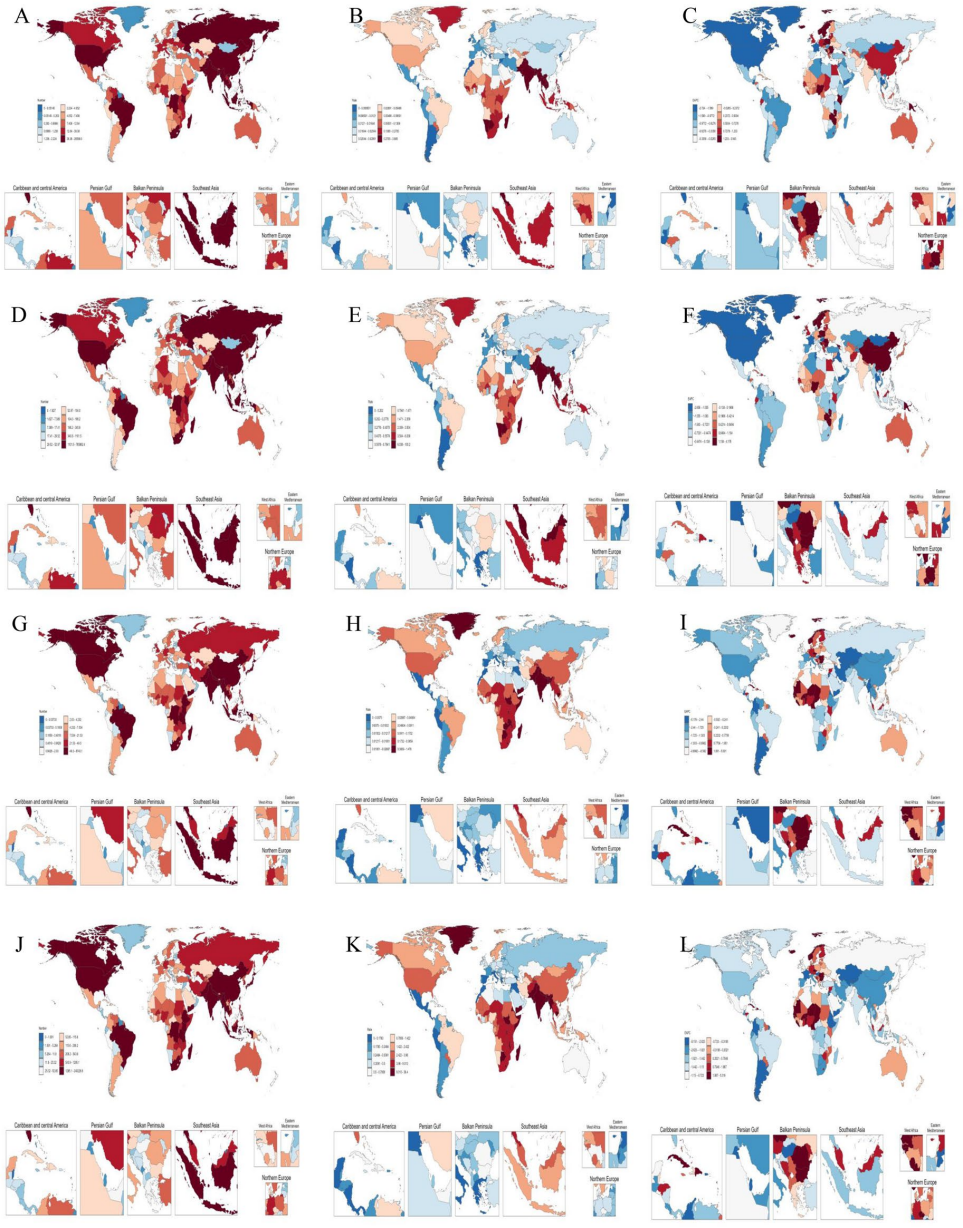
Global burden of chewing tobacco-attributable lip and oral cavity cancer and esophageal cancer for both sexes in 204 countries and territories in 2021. Death numbers in lip and oral cavity cancer (A) and esophageal cancer (G); Age-standardized mortality rates (ASMRs, per 100,000 population) for lip and oral cavity cancer (B) and esophageal cancer (H); Estimated annual percentage change (EAPC) in ASMRs from 2000 to 2021 for lip and oral cavity cancer (C) and esophageal cancer (I); Disability-adjusted life years (DALYs) in lip and oral cavity cancer (D) and esophageal cancer (J); Age-standardized DALY rates (ASDRs, per 100,000 population) for lip and oral cavity cancer (E) and esophageal cancer (K); EAPC of ASDRs from 2000 to 2021 for lip and oral cavity cancer (F) and esophageal cancer (L). Blue indicates statistically significant declines, and red indicates statistically significant increases.

Across 204 countries and territories, the burden of chewing tobacco-attributable cancers demonstrated marked geographic heterogeneity. Lip and oral cavity cancer was primarily concentrated in South Asia and parts of the Pacific, where both deaths and DALYs were highest, including India, Pakistan, and Bangladesh (Figure 1A, D). Age-standardized rates were also elevated in these regions, whereas most countries in Europe and the Americas generally remained at lower levels (Figure 1B, E). By contrast, esophageal cancer displayed a distinct belt” of high burden across East Africa and South Asia, including Comoros, Madagascar, Nepal, and Bangladesh, whether in terms of numbers or age-standardized rates of deaths and DALYs, with most high-income countries showing much lower rates (Figures 1G, H, J, and K)).From 2000 to 2021, lip and oral cavity cancer showed rising trends in a substantial proportion of settings (Figure 1C, F), whereas declines in ASMRs and ASDRs for esophageal cancer were observed in the majority of countries (Figure 1I, L).

China ranked among the top contributors in absolute numbers, within the top ten for lip and oral cavity cancer deaths and DALYs (Figure 1A, D) and second for esophageal cancer deaths (2,442.0) (Figure 1G) and DALYs (65,752.7) (Figure 1J) in 2021. Although the standardized burden of lip and oral cavity cancer in China remains relatively low (ASMR 0.018, ASDR 0.510) (Figure 1B, E), its increase has been more rapid than in many other countries (ASMR: EAPC 0.978, ASDR: EAPC 1.108) (Figure 1C, F). Conversely, the age-standardized burden of esophageal cancer in China decreased markedly (ASMR: EAPC −2.223, ASDR: EAPC −2.548) (Figure 1I, L), consistent with prevention efforts.

### Temporal and sex-specific analysis of chewing tobacco–attributable deaths and DALYs numbers and rates for lip and oral cavity cancer and esophageal cancer worldwide and in China from 2000 to 2021

**Figure 2.**
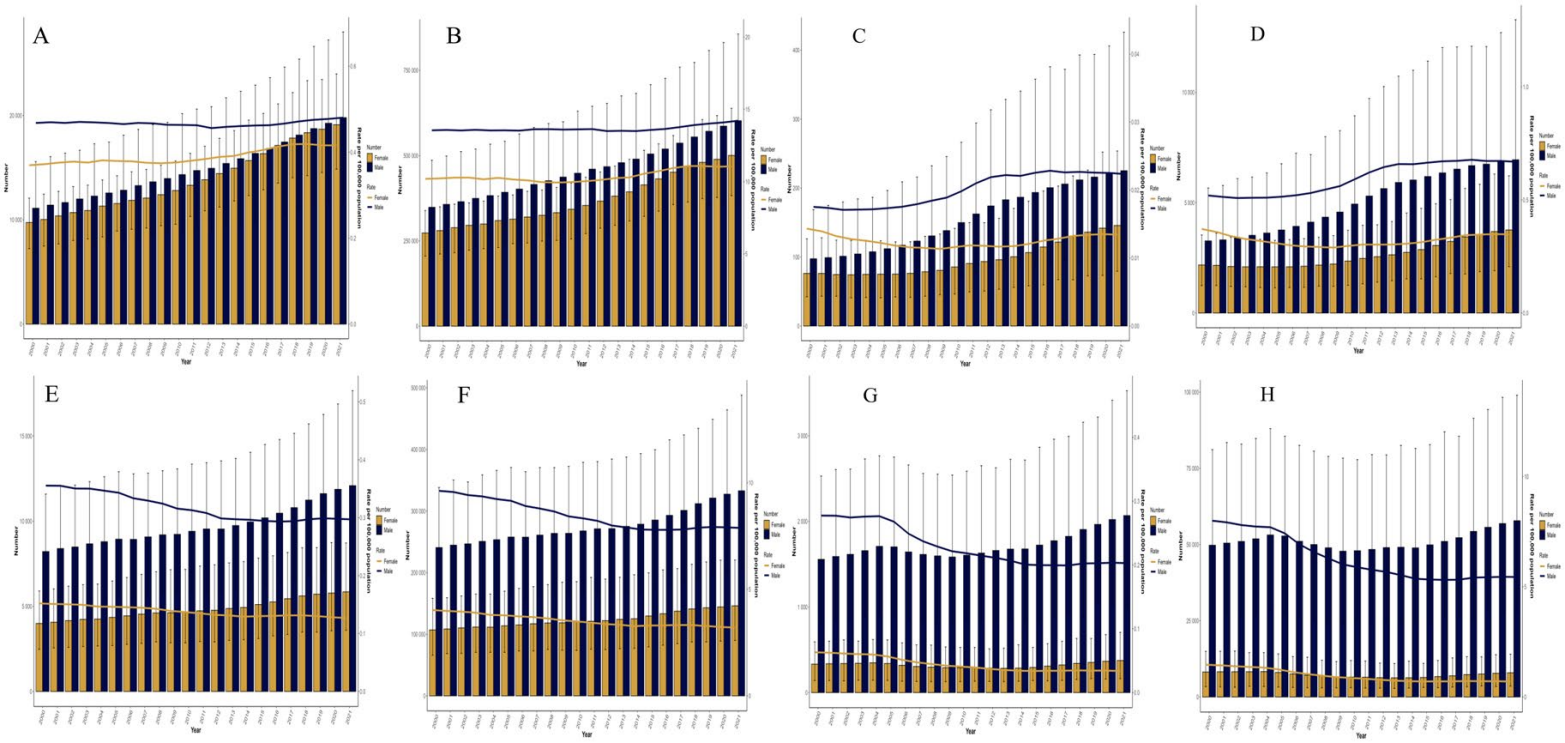
Temporal and sex-specific analysis of chewing tobacco-attributable deaths, DALYs numbers, and rates for lip and oral cavity cancer and esophageal cancer worldwide and in China from 2000 to 2021. Temporal and sex-specific analysis of chewing tobacco-attributable death numbers and rates of lip and oral cavity cancer (A) and esophageal cancer (E) and DALYs numbers and rates of lip and oral cavity cancer (B) and esophageal cancer (F) worldwide from 2000 to 2021. Temporal and gender-specific analysis of chewing tobacco-attributable death numbers and rates of lip and oral cavity cancer (C) and esophageal cancer (G) and DALYs numbers and rates of lip and oral cavity cancer (D) and esophageal cancer (H) in China from 2000 to 2021. Bars represent absolute numbers (left y-axis), and lines represent age-standardized rates per 100,000 population (right y-axis). Dark blue indicates males, and yellow indicates females.

From 2000 to 2021, globally, chewing tobacco-attributable lip and oral cavity cancer deaths increased from 20,846.9 to 38,903.6 (+86.6%), while DALYs increased from 621,796.4 to 1,102,818.4 (+77.4%). Both sexes experienced increases, but the rise was more pronounced among females: female deaths increased by +96.3% compared with +78.1% in males (Figure 2A), and female DALYs grew by +83.1% versus +72.8% in males (Figure 2B). Accordingly, the male-to-female ratio of deaths declined modestly, from 1.12 to 1.03 for deaths and from 1.26 to 1.20 for DALYs. For esophageal cancer, globally, deaths increased from 12,214.7 to 17,939.5 (+46.9%) and DALYs from 347,853.9 to 479,032.7 (+37.7%). Despite these absolute increases, the male burden remained consistently higher, with male deaths and DALYs about twice those of females throughout the period (Figure 2E, F). The male-to-female ratio showed little change, remaining around 2.0.

In China, lip and oral cavity cancer showed a steeper rise. Deaths surged from 173.7 to 370.9 (+113.5%), and DALYs from 5,445.9 to 10,708.8 (+96.6%). The increase was disproportionately greater among males: male deaths increased by +131.0% versus +91.2% in females (Figure 2C), and male DALYs increased by +112.4% versus +73.0% in females (Figure 2D). As a result, the male-to-female ratio exhibited a non-linear trajectory, the deaths ratio rose from 1.20 in 2000 to a maximum of 1.82 in 2013, followed by a decline to 1.47 in 2021, while the DALYs ratio increased from 1.41 in 2000 to 2.15 in 2013, subsequently decreasing to 1.76 in 2021. For esophageal cancer in China, chewing tobacco–attributable deaths increased from 1,888.1 to 2,442.0 (+29.3%), and DALYs rose from 57,972.5 to 65,752.7 (+13.4%). The male-to-female ratio for deaths increased from 4.41 in 2000 to a peak of approximately 5.66 in 2013, and remained elevated at 5.27 by 2021 (Figure 2G). For DALYs, the male-to-female ratio rose from 5.72 in 2000 to a maximum of about 7.52 in 2013, followed by a modest decline to 6.96 in 2021 (Figure 2H). Overall, these patterns indicate a marked amplification of sex disparities in chewing tobacco–attributable esophageal cancer burden in China over time.

### Age- and sex-specific burdens of chewing tobacco–attributable deaths and DALYs numbers and rates for lip and oral cavity cancer and esophageal cancer worldwide and in China in 2021

**Figure 3.**
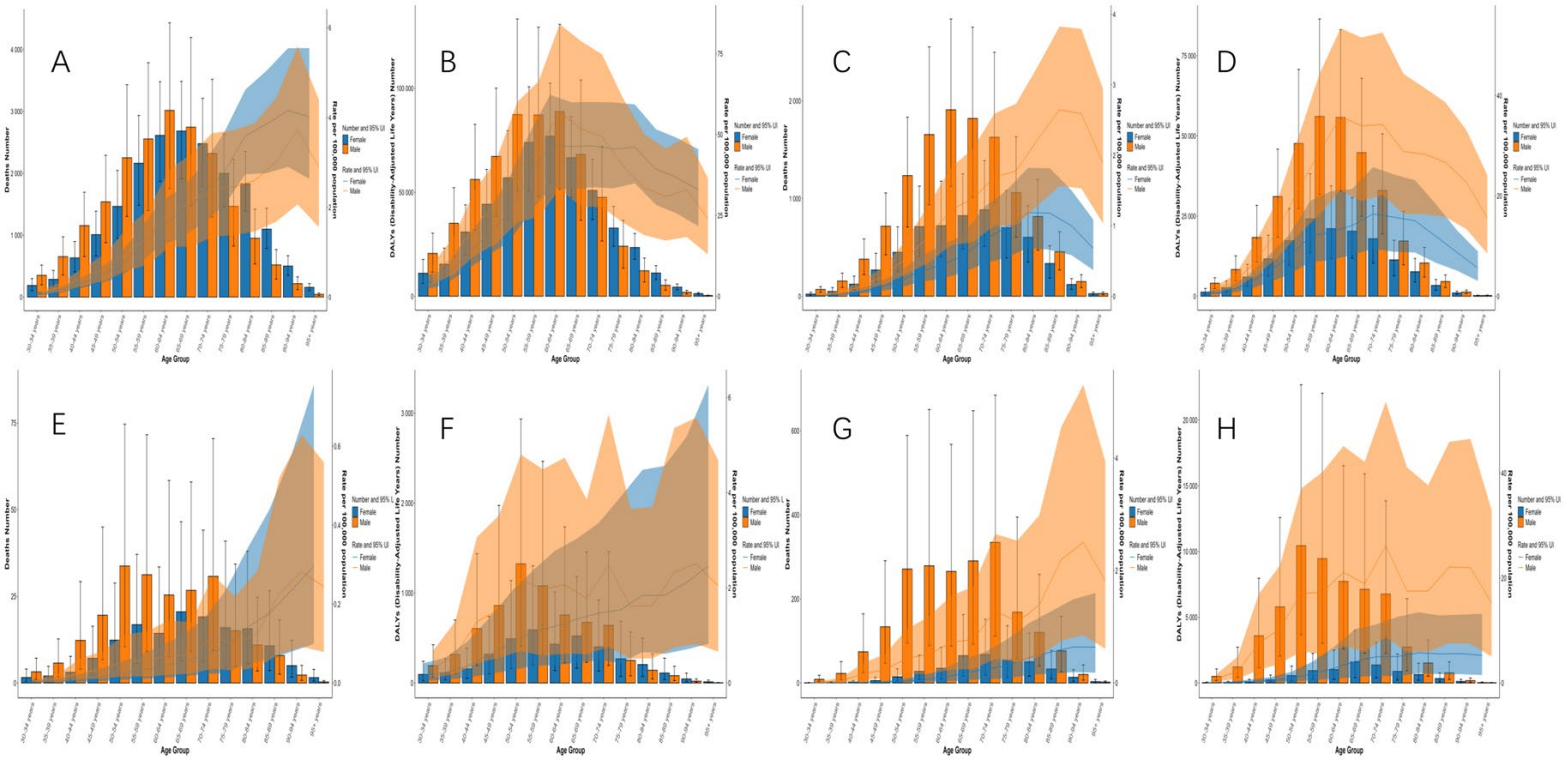
Age- and sex-specific burdens of chewing tobacco–attributable deaths and DALYs numbers and rates of lip and oral cavity cancer and esophageal cancer worldwide and in China in 2021. Global numbers and rates of chewing tobacco–attributable deaths (A) and DALYs (B) of lip and oral cavity cancer and chewing tobacco–attributable deaths (C) and DALYs (D) of esophageal cancer by age and sex in 2021. China numbers and rates of chewing tobacco–attributable deaths (E) and DALYs (F) of lip and oral cavity cancer and chewing tobacco–attributable deaths (G) and DALYs (H) of esophageal cancer, by age and sex, in 2021. Bars represent absolute numbers (left y-axis), and lines represent age-standardized rates per 100,000 population (right y-axis). Orange indicates males, and blue indicates females. All metrics are presented with 95% uncertainty intervals. Age-standardized rate was calculated using the GBD global standard population.

In 2021, the global burden of chewing tobacco-attributable lip and oral cavity cancer demonstrated marked age- and sex-specific patterns. Deaths were concentrated in older age groups, peaking at 60–64 years among males and 65–69 years among females, with 3,017.8 male deaths and 2,683.5 female deaths worldwide (Figure 3A). Corresponding peaking DALYs in 60–64 age groups with 88,807.8 in males and 77,004.6 in females (Figure 3B). The male-to-female ratio reduced progressively with age, peaking at approximately 2.2 for both deaths and DALYs in the 35–39 age group, before fluctuating slightly in the oldest age groups. For esophageal cancer, global deaths showed a more even age distribution, with deaths peaking in the 65–69 age group (Figure 3C), which approached 1,820.6 in males and 826.0 in females, while DALYs peaked in the 55–59 age group, which approached 56,066.6 in males and 24,140.9 in females (Figure 3D). The male-to-female ratio reduced progressively with age, peaking at approximately 2.23 for deaths and 3.70 for DALYs in the 40–44 age group, before fluctuating slightly in the oldest age groups.

In China, the absolute burden of lip and oral cavity cancer was lower but exhibited a sharper male predominance (Figure 3E, F). Deaths peaked at 30.76 among males and 19.06 among females in the 70–74 age groups, while DALYs approached 1,080.4 in males and 590.5 in females in 55–59 age groups. The male-to-female ratio reduced progressively with age, peaking at approximately 3.77 for deaths and at 3.70 for DALYs in the 40–44 age group, before fluctuating slightly in the oldest age groups. For esophageal cancer, the sex disparity was markedly more pronounced. Deaths peaked in the 70–74 age group, approaching 334.6 in males and 67.6 in females (Figure 3G), with DALYs peaking in the 50–54 age group, reaching 10,443.9 in males and 563.4 in females (Figure 3H). The male-to-female ratio was exceptionally high, peaking at approximately 32.1 for deaths and at 31.8 in DALYs in the 40–44 age group, before declining and fluctuating slightly in the oldest age groups.

### Decomposition analysis of chewing tobacco–attributable deaths and DALYs for lip and oral cavity cancer and esophageal cancer worldwide and in China in 2021

**Figure 4.**
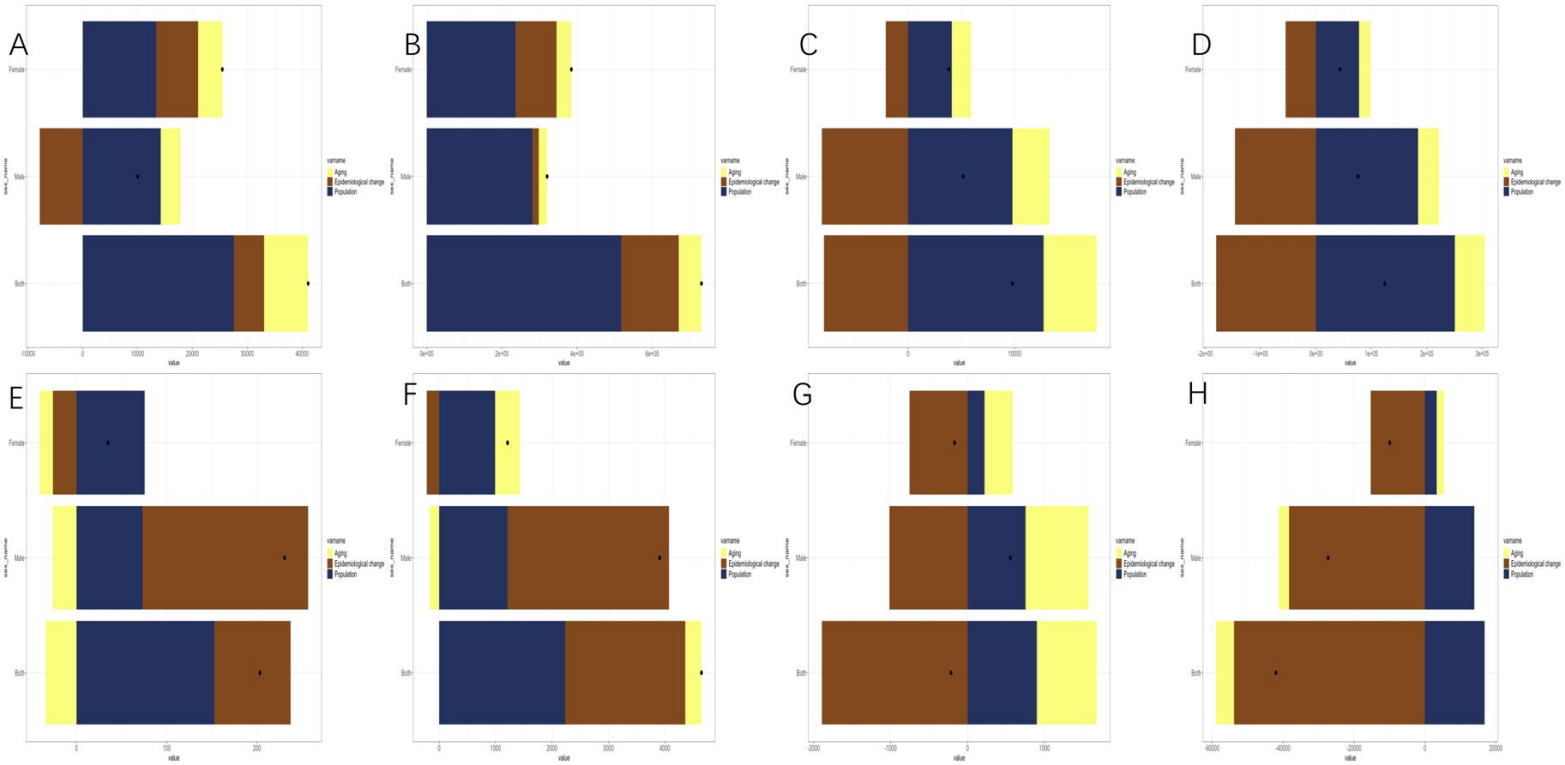
Decomposition of chewing tobacco–attributable lip and oral cavity cancer and esophageal cancer deaths and DALYs, by sex, 2000–2021, worldwide and in China. (A–D) show global estimates: (A) deaths from lip and oral cavity cancer, (B) DALYs from lip and oral cavity cancer, (C) deaths from esophageal cancer, and (D) DALYs from esophageal cancer. (E–H) present corresponding estimates for China: (E) deaths from lip and oral cavity cancer, (F) DALYs from lip and oral cavity cancer, (G) deaths from esophageal cancer, and (H) DALYs from esophageal cancer. Results are stratified by sex (female, male, or both). Stacked bars depict the contributions of population aging, population growth, and epidemiological change to the net change, while black dots indicate the overall observed change. In strata with very small absolute changes, the decomposition may yield numerically extreme percentage contributions; these values reflect mathematical instability rather than disproportionately large absolute effects and should therefore be interpreted with caution.

Globally, from 2000 to 2021, the overall difference in chewing tobacco–attributable deaths for lip and oral cavity cancer was +41,100.2 (Figure 4A). Decomposition analysis showed that aging contributed +8,031.0 (19.5%), population growth +27,563.2 (67.1%), and epidemiological change +5,506.1 (13.4%). When stratified by sex, male deaths rose by +10,018.8, with aging (+3,642.7, 36.4%) and population growth (+14,217.5, 141.9%) as major drivers, partly offset by unfavorable epidemiological change (–7,841.4, −78.3%). Female deaths increased more steeply (+25,463.7), with aging (+4,428.4, 17.4%), population growth (+13,390.6, 52.6%), and epidemiological change (+7,644.6, 30.0%) all contributing positively. Corresponding DALYs overall difference is +731,036.6 (Figure 4B), mainly due to population growth (+517,995.8, 70.9%) and epidemiological change (+152,483.9, 20.9%), with a smaller contribution from aging (+60,557.0, 8.3%). Among males, DALYs differed overall by 320,436.0, predominantly explained by population growth (+281,284.4, 87.78%), while females experienced a larger absolute increase (+384,940.7), with a greater contribution from epidemiological change (+108,902.9, 28.3%) compared to males (+16,898.7, 5.3%).

For esophageal cancer globally, deaths differed overall by +9,789.7 (Figure 4C), attributable to population aging (+4,969.9, 50.8%) and population growth (+12,709.3, 129.8%), partly offset by substantial favorable epidemiological change (–7,889.4, −80.6%). Among males, the overall difference in deaths was +5,177.2, driven by aging (+3,474.4, 67.1%) and population growth (+9,786.0, 189.0%), but substantially offset by epidemiological improvement (–8,083.2, −156.1%). Among females, deaths differed overall by +3,817.4, with demographic factors dominating (aging: +1,806.4; population growth: +4,101.5), partly counterbalanced by epidemiological improvements (–2,090.5, −54.8%). Corresponding DALYs overall difference modestly by +124,086.3 (Figure 4D), where population growth (+250,659.4, 202.0%) was counterbalanced by negative epidemiological change (–180,235.8, −145.3%). Male DALYs overall difference by +76,052.1 were almost entirely demographic-driven, while female DALYs also showed moderate gains by +43,253.7, both largely mitigated by epidemiological decline (males: −146,219.951, −192.3%, females: −54,929.864, −127.0%).

In China, decomposition patterns differed substantially. For lip and oral cavity cancer, deaths differed overall by +203.1 (Figure 4E), with positive contributions from epidemiological change (+84.4, 41.6%) and population growth (+152.9, 75.3%), while population aging slightly reduced the burden (–34.2, −16.8%). Among males, deaths differed overall by +230.5, primarily driven by population growth (+73.2, 31.8%) and unfavorable epidemiological shifts (+183.5, 79.6%), despite a small negative aging effect (–26.2, −11.4%). Among females, deaths overall difference modestly by +34.9, largely attributable to population growth (+75.6, 216.7%). DALYs from lip and oral cavity cancer in China differed overall by +4,648.3 (Figure 4F), mainly driven by epidemiological change (+2,127.0, 45.8%) and population growth (+2,234.3, 48.1%). Among males, DALYs overall difference by +3,907.7, predominantly due to epidemiological change (2,859.4, 73.2%), despite a small negative aging contribution (–165.6, −4.2%). Among females, DALYs showed an overall difference by +1,212.92, largely explained by population aging (+441.6, 36.4%) and population growth (+991.8, 81.8%), with a small negative epidemiological effect (−220.5, −18.2%). By contrast, chewing tobacco–attributable esophageal cancer in China showed an overall decline. Deaths differed overall by −215.2 (Figure 4G). Although the decomposition of deaths yields numerically extreme percentage contributions (for example, epidemiological change approximately +880%), these largely reflect mathematical instability due to small baseline counts rather than disproportionately large absolute effects and should therefore be interpreted with caution. Corresponding DALYs fell by −42,035.4 (Figure 4H), driven by substantial favorable epidemiological change (−53,823.7, 128.0%), which more than offset demographic pressures (aging: −5,035.302, 11.98%, population growth: +16,823.523, −40.02%). Male DALYs decreased by −27,303.1, and female DALYs by −9916.8, both primarily attributable to epidemiological improvements.

### Age-period-cohort analysis of deaths and DALYs rate for chewing tobacco–attributable lip and oral cavity cancer and esophageal cancer worldwide and in China

**Figure 5.**
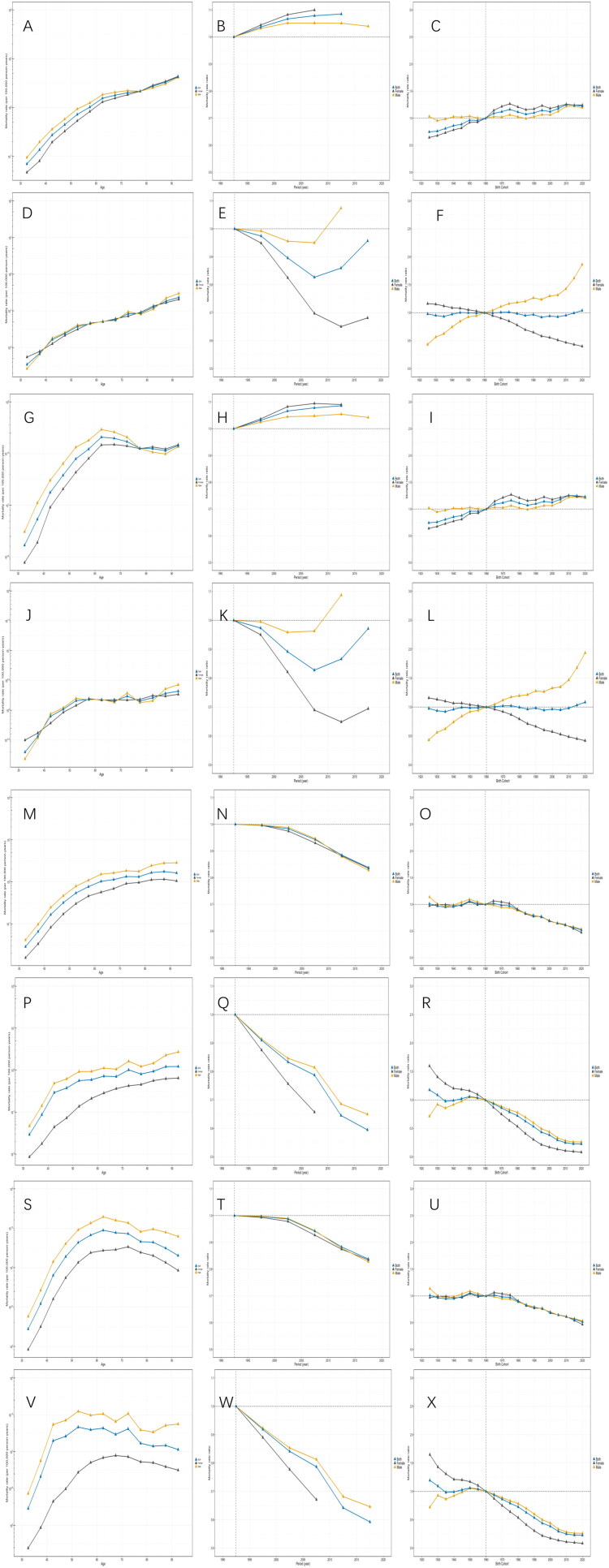
Age-period-cohort analysis of chewing tobacco–attributable deaths and DALYs for lip and oral cavity cancer and esophageal cancer worldwide and in China. (A–C) show age, period, and cohort effects for deaths from lip and oral cavity cancer worldwide, and (G–I) show the corresponding effects for DALYs. (D–F) and (J–L) present age, period, and cohort effects for deaths and DALYs from lip and oral cavity cancer in China, respectively. (M–O) show age, period, and cohort effects for deaths from esophageal cancer worldwide, and (S–U) show the corresponding effects for DALYs. (P–R) and (V–X) present age, period, and cohort effects for deaths and DALYs from esophageal cancer in China, respectively. Results are stratified by sex, with dark blue representing both sexes combined, dark gray representing females, and orange representing males. Age-specific effects are presented as rates per 100,000 population, whereas period and cohort effects are presented as rate ratios (RRs). Vertical dashed lines indicate the reference period (1990–1995) for period effects and the reference birth cohort (1960) for cohort effects.

Globally, the age effect of deaths (Figure 5A) and DALYs (Figure 5G) in lip and oral cavity cancer increased steadily with advancing age, with consistently higher relative risks among males than females. Corresponding period effect of deaths (Figure 5B) and DALYs (Figure 5H) showed a mild upward trend between 1990 and 2020. The cohort effect demonstrated elevated risks of deaths (Figure 5C) and DALYs (Figure 5I) among birth cohorts from approximately 1925 to 1960, followed by an increase towards unity in more recent cohorts. For esophageal cancer, the age effect of deaths (Figure 5M) also rose with age, but DALYs (Figure 5S) exhibited a Inverted V-shape”, which reaches a peak and declines slowly. Corresponding period effects of deaths (Figure 5N) and DALYs (Figure 5T) showed a persistent decline from 1990 to 2020. Similarly, the cohort effect of deaths (Figure 5O) and DALYs (Figure 5U) decreased steadily and similarly between males and females, with ratio rates (RRs) of deaths and DALYs declining from 1.01 in the 1925 birth cohort to 0.52 in the 2020 birth cohort.

In China, the age effect of deaths (Figure 5D) and DALYs (Figure 5J) in lip and oral cavity cancer increased steadily with age, with similar risk patterns observed in males and females. Corresponding period effect of deaths (Figure 5E) and DALYs (Figure 5K) exhibited a U-shaped’ pattern, particularly among males. The cohort effect of deaths (Figure 5F) and DALYs (Figure 5L) diverged markedly by sex: among males, birth cohorts after 1960 showed rising risks, with RRs for deaths rising to 1.86 and for DALYs to 1.94 in the 2020 birth cohort, whereas female cohorts showed a consistent decline, decreasing to 0.40 for deaths and 0.42 for DALYs in the 2020 birth cohort. For esophageal cancer, the age effect of deaths (Figure 5P) increased with age, but the age effect for DALYs (Figure 5V) reached a peak, fluctuated, and declined slowly. Corresponding period effect of deaths (Figure 5Q) and DALYs (Figure 5W) declined more sharply than the global average, with females more sharply than males. The cohort effect of deaths (Figure 5R) and DALYs (Figure 5X) also showed a pronounced decrease, with RRs for deaths decreasing from 1.17 in 1925 birth cohorts to 0.23 in the 2020 birth cohorts, and RRs for DALYs decreasing from 1.19 in 1925 birth cohorts to 0.23 in 2020 birth cohorts. The above detailed results are shown in Table 3.

**Table 3.** Net drift estimates of age-standardized mortality rates (ASMRs) and age-standardized disability-adjusted life year rates (ASDRs) for lip and oral cavity cancer and esophageal cancer attributable to chewing tobacco worldwide and in China.

		Global				China			
		ASMRs		ASDRs		ASMRs		ASDRs	
		Net drift (% per year)	(95% CI)	Net drift (% per year)	(95% CI)	Net drift (% per year)	(95% CI)	Net drift (% per year)	(95% CI)
Lip and oral cavity cancer	Both	−2.058	(−4.247, 0.182)	−2.117	(−2.496, −1.737)	−3.195	(−13.102, 7.843)	−3.278	(−5.042, −1.481)
	Male	−2.389	(−4.385, −0.351)	−2.455	(−2.807, −2.102)	−2.235	(−11.323, 7.784)	−2.283	(−3.946, −0.591)
	Female	−1.838	(−4.321, 0.708)	−1.906	(−2.324, −1.486)	−4.306	(−15.984, 8.995)	−4.371	(−6.382, −2.316)
Esophageal cancer	Both	−3.161	(−6.719, 0.533)	−3.202	(−3.784, −2.616)	−4.984	(−9.385, −0.370)	−5.037	(−5.753, −4.316)
	Male	−3.209	(−6.156, −0.168)	−3.265	(−3.745, −2.783)	−4.632	(−7.937, − 1.209)	−4.680	(−5.221, −4.137)
	Female	−3.183	(−8.000, 1.886)	−3.222	(−3.998, −2.441)	−6.364	(−18.620, 7.739)	−6.455	(−8.340, −4.532)

The table presents net drift estimates (% per year) with 95% confidence intervals (CIs) derived from age–period–cohort analyses, summarizing overall annual percentage changes in ASMRs and ASDRs for lip and oral cavity cancer and esophageal cancer attributable to chewing tobacco. Estimates are shown globally and for China, stratified by sex.

### BAPC projections of chewing tobacco–attributable lip and oral cavity cancer and esophageal cancer worldwide and in China from 2000 to 2036

**Figure 6.**
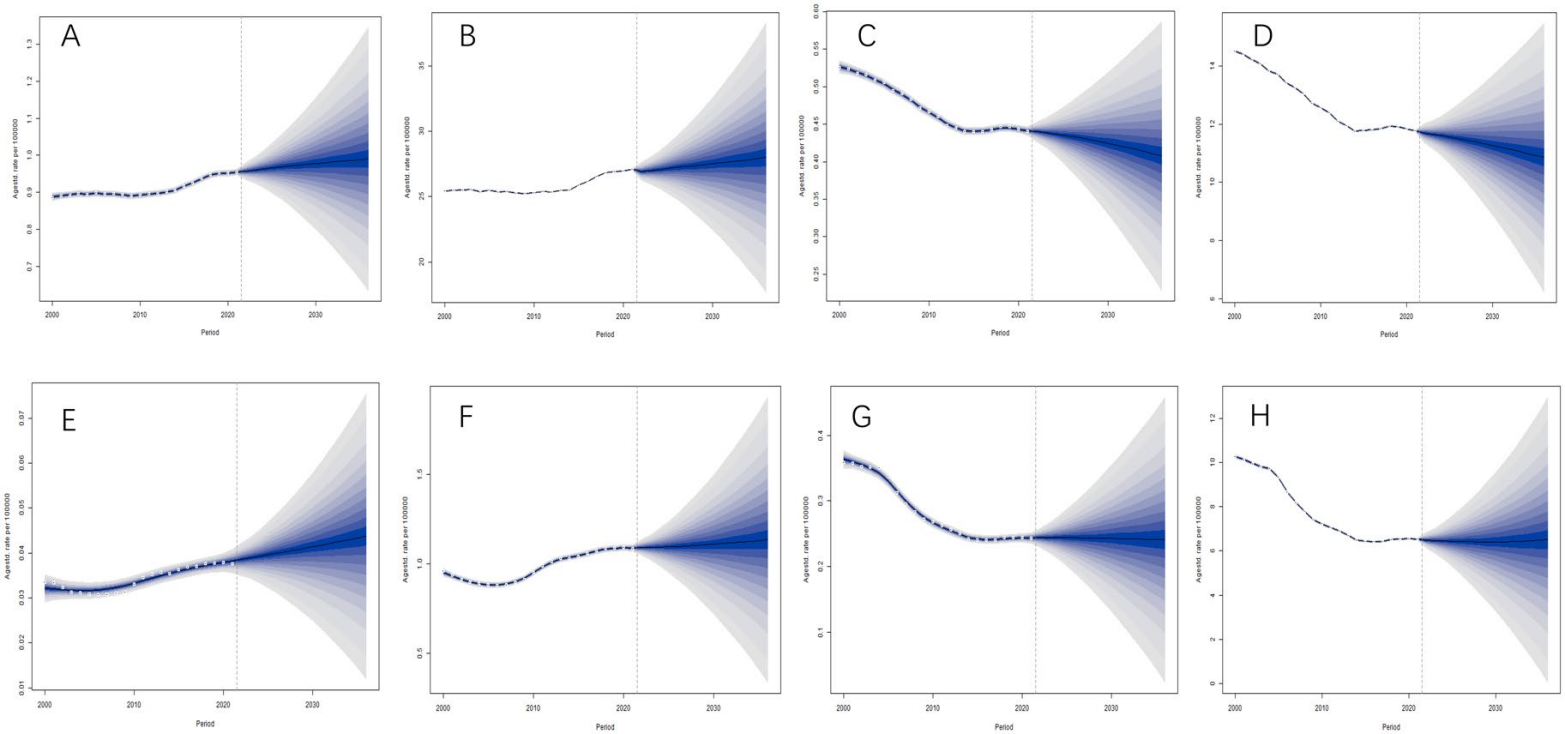
BAPC projections of ASMRs and ASDRs for chewing tobacco–attributable lip and oral cavity cancer and esophageal cancer worldwide and in China from 2000 to 2036. (A and B) show ASMRs and ASDRs for lip and oral cavity cancer worldwide, and (C and D) show ASMRs and ASDRs for esophageal cancer worldwide. (E and F) show ASMRs and ASDRs for lip and oral cavity cancer in China, and (G and H) show ASMRs and ASDRs for esophageal cancer in China. All rates are presented per 100,000 population. Solid lines represent mean estimates, and shaded areas denote 95% uncertainty intervals. All metrics were age-standardized to the GBD global standard population. Vertical dashed lines indicate the transition from historical data (2000–2021) to future projections (2022–2036).

Globally, the age-standardized burden of chewing tobacco–attributable lip and oral cavity cancer showed a modest upward trend, with ASMRs increasing from 0.888 per 100,000 in 2000 to 0.955 in 2021 (+7.5%) and are projected to reach 0.990 (95% UI 0.634–1.347) by 2036 (+3.7% from 2021) (Figure 6A). Similarly, ASDRs rose from 25.433 in 2000 to 27.094 in 2021 (+6.5%) and are projected to increase to 27.994 (95% UI 17.727–38.261) by 2036 (+3.3%) (Figure 6B). In contrast, the global burden of chewing tobacco–attributable esophageal cancer declined over the study period. The ASMRs decreased from 0.527 per 100,000 in 2000 to 0.441 in 2021 (−16.3%), with a further projected decline to 0.408 (95% UI 0.228–0.587) by 2036 (−7.5% from 2021) (Figure 6C). Correspondingly, the ASDRs declined from 14.504 in 2000 to 11.772 in 2021 (−18.8%) and are projected to reach 10.856 (95% UI 6.248–15.464) by 2036 (−7.8%) (Figure 6D).

In China, chewing tobacco–attributable lip and oral cavity cancer demonstrated a sustained increase. ASMRs rose from 0.032 per 100,000 in 2000 to 0.038 in 2021 (+18.9%) and are projected to increase to 0.044 (95% UI 0.012–0.075) by 2036 (+14.4%) (Figure 6E). ASDRs increased from 0.951 in 2000 to 1.089 in 2021 (+14.5%) and are projected to reach 1.135 (95% UI 0.338–1.932) by 2036 (+4.3%) (Figure 6F). Conversely, chewing tobacco–attributable esophageal cancer in China exhibited a more pronounced decline than the global trend. The ASMRs decreased from 0.363 per 100,000 in 2000 to 0.244 in 2021 (−32.9%) and is projected to plateau at 0.241 (95% UI 0.024–0.458) by 2036 (−1.1%) (Figure 6G). Similarly, the ASDRs declined from 10.264 in 2000 to 6.534 in 2021 (−36.3%) and is projected to remain largely stable at 6.508 (95% UI 0.075–12.941) by 2036 (−0.4%) (Figure 6H). Overall, BAPC projections were accompanied by relatively wide uncertainty intervals, reflecting both uncertainty in extrapolating historical trends and the sensitivity of future burden estimates to potential changes in chewing tobacco–attributable risk.

## Discussion

This study provides a comprehensive assessment of the global and national burden of chewing tobacco-attributable lip and oral cavity cancer and esophageal cancer from 2000 to 2021, integrating age–period–cohort analysis, decomposition analysis, and BAPC projections. The findings reveal distinct temporal, age-specific, site-specific, sex-specific, and cancer-specific trajectories, reflecting both progress and persistent gaps in chewing tobacco control and cancer prevention.

Temporal and age-specific patterns demonstrated a similarity to the overall trend. Globally, chewing tobacco-attributable deaths and DALYs from lip and oral cavity cancer nearly doubled, whereas esophageal cancer showed absolute number growth despite continuous declines in age-standardized rates. In China, the overall trend is similar between cancers: chewing tobacco-attributable lip and oral cavity cancer and esophageal cancer deaths and DALYs absolute number growth but age-standardized rates of esophageal cancer dropped significantly. DALYs for both cancers worldwide and in China peaked at younger ages than deaths, indicating that functional health loss precedes mortality.

Comparisons between global and China-specific trends underscore asymmetrical progress. China experienced a greater relative increase in lip and oral cancer burden than the global average, albeit from a much lower baseline, and experienced a more pronounced decrease in standardized burden of esophageal cancer compared with global trends. This divergence suggests that, while esophageal cancer control in China has been relatively successful, preventive efforts for lip and oral cancer remain insufficient. In China, both lip and oral cavity cancer and esophageal cancer exhibited earlier DALY peaks (55–59 and 50–54 years, respectively) but later death peaks (70–74 years) than globally, indicating that morbidity occurs at younger ages while mortality is delayed. This pattern may reflect relatively improved survival due to clinical treatment despite persistent early-life exposures to risk factors. Decomposition analysis further clarified the drivers of these trends. Globally, chewing tobacco-attributable deaths and DALYs were largely driven by demographic factors, including population growth and population aging. In China, population growth was the dominant contributor, while population aging partially offset the overall demographic effect, suggesting that favorable epidemiological changes may already partially counterbalance the impact of population aging. BAPC projections to 2036 indicated that the burden of lip and oral cancer will continue to rise, with the upward trend more evident in China despite its lower baseline, whereas chewing tobacco-attributable esophageal cancer is projected to decline further worldwide and particularly in China. From a methodological perspective, although the BAPC projections appear plausible overall, the width of the uncertainty intervals varies across countries and cancer sites. In settings such as China, relatively wider uncertainty intervals for lip and oral cavity cancer reflect greater variability in historical trends and uncertainty in chewing tobacco exposure trajectories, which are propagated through the Bayesian framework. Future research may further enhance projection accuracy by incorporating advanced modelling strategies—such as multiplex network-based approaches—to capture complex interactions among multiple risk factors and demographic processes, complementing traditional APC and BAPC models [24–26].

Sex disparities remained substantial but followed distinct patterns globally and in China. Globally, the burden of chewing tobacco–attributable lip and oral cavity cancer increased faster among females, whereas the burden of esophageal cancer declined faster among males, resulting in a narrowing sex gap for lip and oral cavity cancer but persistent male predominance for esophageal cancer (male-to-female ratio approximately 2.0). In contrast, in China, the burden of lip and oral cavity cancer increased faster among males, while esophageal cancer declined faster among females, leading to a widening male predominance. Male-to-female ratios reached approximately 3.8 for lip and oral cavity cancer and >30 for esophageal cancer deaths in the 40–44 age groups. This pronounced mid-adulthood peak likely reflects substantial sex differences in smokeless tobacco exposure in China, where chewing tobacco and related practices are predominantly concentrated among men and often initiated at younger ages, resulting in greater cumulative exposure by mid-adulthood. Notably, the apparent decline in esophageal cancer among females should be interpreted with caution, as relatively low case counts may lead to greater year-to-year variability and amplify apparent trend magnitudes.

In addition, lip and oral cavity cancer show sex-divergent epidemiological characteristics: globally, female epidemiological risks increased after accounting for demographic effects, whereas in China, epidemiological risks increased among males but decreased among females. Cohort effects also diverged markedly by sex: globally, relative risks for deaths and DALYs increased in males and females from 1925 birth cohorts to 2020 birth cohorts, whereas in China male cohorts showed rising risks, while female cohorts showed steady declines. These findings indicate persistently elevated risk among Chinese men, particularly for esophageal cancer, and underscore the need for male-targeted chewing tobacco control and oral health promotion.

Beyond demographic dynamics, cross-national differences in disease burden are likely shaped by broader socioeconomic development and health system capacity. Countries with higher Human Development Index (HDI) levels generally benefit from stronger tobacco control policies, wider access to cancer screening, and improved treatment, which may contribute to the declining burden of esophageal cancer. In contrast, in lower- and middle-HDI settings, persistent exposure to multiple risk factors—including chewing tobacco, alcohol consumption, and betel quid chewing—combined with limited preventive and diagnostic services may underlie the continued rise in lip and oral cavity cancer burden.

The contrasting trajectories of the two cancers illustrate heterogeneous chewing tobacco impacts along the upper digestive tract. The sustained decline in esophageal cancer is primarily driven by favorable epidemiological changes, as demonstrated by decomposition analysis, and is consistent with strengthened chewing tobacco control alongside broader improvements in risk-factor management and healthcare access. In contrast, the continued rise in lip and oral cavity cancer highlights persistent gaps in prevention strategies and the complexity of risk exposures.

Accumulating evidence indicates that betel quid, tobacco, and alcohol exert synergistic carcinogenic effects within the lip and oral cavity, partly through downregulation of antioxidant defenses and consequent enhancement of reactive oxygen species formation [27]. Accordingly, the increasing burden of lip and oral cavity cancer cannot be attributed to tobacco exposure alone and underscores the need for comprehensive prevention approaches targeting multiple co-exposures. Integrated interventions addressing tobacco use, alcohol consumption, betel quid chewing, and oral hygiene should therefore be prioritized through strengthened prevention programs and targeted public health education.

From a public health perspective, these findings call for a shift from uniform chewing tobacco control toward age-specific, site-specific, sex-specific, and cancer-specific integrated prevention strategies. Sustaining esophageal cancer gains requires continued investment in screening and treatment access, whereas reducing the burden of lip and oral cancer will depend on comprehensive approaches linking cessation of tobacco, alcohol, and betel quid chewing with oral health education and early detection within primary care. Globally, precision tobacco control aligned with demographic and exposure profiles should become central to future cancer prevention frameworks.

In conclusion, this study identifies a dual epidemiological transition: a sustained decline in chewing tobacco-attributable esophageal cancer, contrasted with a growing burden of lip and oral cavity cancer, particularly among Chinese men. Further reductions in tobacco-related cancer burden will depend not only on maintaining chewing tobacco control but also on addressing co-exposures and strengthening oral health education–focused prevention tailored by age, site, sex, and cancer type.

## Conclusions

This study demonstrates divergent trends in chewing tobacco-attributable cancers. From 2000 to 2021, the age-standardized burden of esophageal cancer declined substantially worldwide and even more sharply in China, reflecting the benefits of long-term chewing tobacco control, early detection, and improved treatment. In contrast, the burden of lip and oral cavity cancer increased steadily, particularly among males and working-age populations in China, with projections indicating further growth to 2036.

These contrasting trajectories reflect both substantial progress and persistent gaps in cancer prevention. The sustained decline in esophageal cancer burden illustrates the effectiveness of sustained public health interventions, whereas the rising burden of lip and oral cavity cancer indicates insufficient control of relevant risk factors. Given the well-established synergistic carcinogenic effects of chewing tobacco with alcohol consumption and betel quid chewing, integrated prevention strategies are warranted. Such strategies should include strengthened control of tobacco, alcohol, and betel quid use, enhanced oral health education, targeted early detection, and precision prevention tailored by age, site, sex, and cancer to sustain gains in esophageal cancer control and mitigate the rising burden of lip and oral cavity cancer.

## Limitations

This study has several limitations. First, our analysis was based on secondary data derived from the Global Burden of Disease (GBD) 2021. Although the GBD framework applies standardized modeling approaches and rigorous quality control procedures, cross-country variation in the quality, completeness, and availability of primary data may affect the accuracy of the estimates and limit opportunities for external validation. In particular, the burden attributable to chewing tobacco, a major form of smokeless tobacco, is likely to be underestimated in many Asian countries because of limited product-specific exposure data and underreporting, as consistently documented in previous GBD studies [6,7]. Second, substantial differences in health system capacity, cancer screening coverage, and tobacco control intensity persist even among countries with similar sociodemographic profiles. Although these contextual factors are closely correlated with socioeconomic development, including the Human Development Index (HDI), the absence of sufficiently detailed country-specific data precluded disentangling their independent contributions to the observed disease burden. Third, future projections were derived from observed historical trends and did not explicitly model alternative scenarios of socioeconomic development. Consequently, potential variations in disease burden trajectories under different Sociodemographic Index (SDI) levels could not be assessed. Finally, the GBD cause hierarchy aggregates certain subsites of lip and oral cavity cancer and esophageal cancer, which may obscure subsite-specific etiological or pathological differences [28].

## Data Availability

The data used in this study are publicly available from the Global Burden of Disease (GBD) Study. All analyses were conducted using standard GBD analytical frameworks. Additional analytical details are available from the corresponding author upon reasonable request.
